# High hopes for cannabinoid agonists in the treatment of rheumatic diseases

**DOI:** 10.1186/1471-2474-15-410

**Published:** 2014-12-04

**Authors:** Caroline A Staunton, Ali Mobasheri, Richard Barrett-Jolley

**Affiliations:** Department of Musculoskeletal Biology, Institute of Ageing and Chronic Disease, University of Liverpool, Liverpool, L69 3GA United Kingdom; The D-BOARD European Consortium for Biomarker Discovery, School of Veterinary Medicine, Faculty of Health and Medical Sciences, University of Surrey, Duke of Kent Building, Guildford, Surrey GU2 7XH UK; Center of Excellence in Genomic Medicine Research (CEGMR), King Fahd Medical Research Center (KFMRC), King Abdul Aziz University, Jeddah, 21589 Saudi Arabia; School of Veterinary Medicine, Faculty of Health and Medical Sciences, University of Surrey, Duke of Kent Building, Guildford, Surrey GU2 7XH UK; Arthritis Research UK Centre for Sport, Exercise and Osteoarthritis, Arthritis Research UK Pain Centre, Medical Research Council and Arthritis Research UK Centre for Musculoskeletal Ageing Research, University of Nottingham, Queen’s Medical Centre, Nottingham, NG7 2UH UK

**Keywords:** Cannabinoids, Cannabis, Chronic pain, Dorsal root ganglia, Ion channels, Fibroblast-like synoviocytes (FLS), JW133, Osteoarthritis (OA), Rheumatoid Arthritis (RA)

## Abstract

There are two well-characterised isoforms of cannabinoid receptor; CB_1_ and CB_2_ and of these CB_2_ is under active investigation as a potential target for treatment of the chronic pain associated with widespread and intractable joint diseases osteoarthritis and rheumatoid arthritis. The recent report by Fukuda *et al* (*BMC Musculoskelet Disord***15:** 275, 2014) in BMC Musculoskeletal Disorders investigates the efficacy of a selective CB_2_ agonist, JW133, in both *in vitro* and *in vivo* models of rheumatoid arthritis and provides encouraging data. The report shows that JW133 inhibits expression of the CCL2 cytokine, osteoclastogenesis and reduces histological indicators of joint degeneration. Each of these could potentially contribute to beneficial analgesic effects in a therapeutic context.

**Electronic supplementary material:**

The online version of this article (doi:10.1186/1471-2474-15-410) contains supplementary material, which is available to authorized users.

## Background

**Figure 1:**
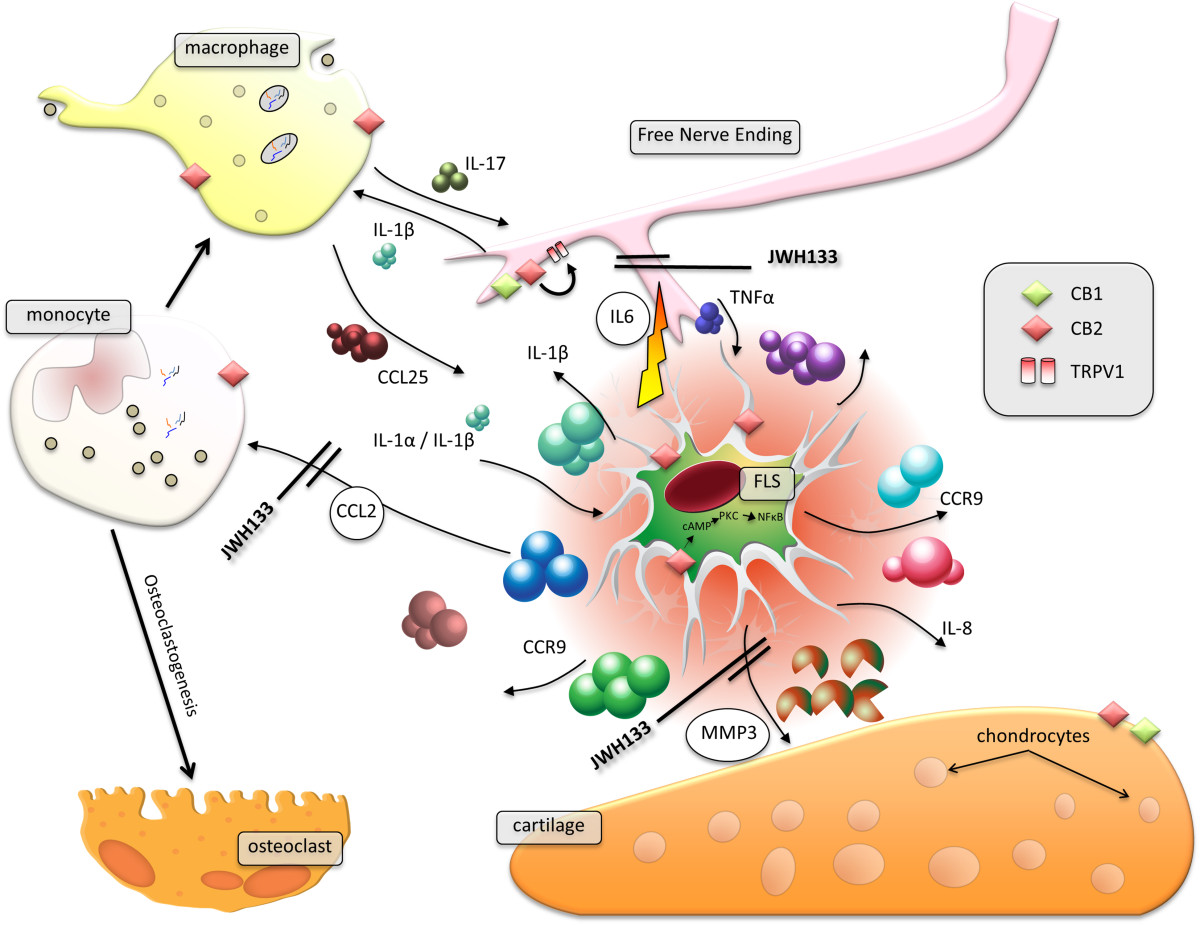
**Cannabinoids have multiple actions in the synovium.** The synovium is a richly vascularised and innervated tissue that becomes inflamed in both RA[12] and OA[13]. Fibroblast-like synovial cells (FLS) lie at the heart of synovial tissue; producing synovial fluid and mediating both pro- and anti-inflammatory properties of the synovium. A number of cytokines act on the FLS and induce release of further cytokines. The CB_2_ receptor is expressed more in the RA synovium than that of the OA joint. Production of CCL2, MMP-3 and IL-6 were all suppressed by the selective CB_2_ agonist JWH133 applied to in TNF-α stimulated FLS. Monocyte osteoclastogenesis was also suppressed. These factors are all important in arthritis. IL-6 mediates pain, MMP-3 mediates cartilage destruction and CCL-2 is a monocyte chemoattractant protein. Monocytes giving rise to osteoclasts (which re-absorb bone) and infiltrating macrophages that perpetuate inflammation. Action of cannabinoids will prove more complex however in complete joints, since CB_2_ receptors interact with TRPV1 and have been reported to increase afferent nerve firing[14], and chondrocytes express both CB_1_ and CB_2_ under some conditions[15].

The cannabinoids are a family of compounds from the plant *Cannabis sativa* L. (*sativa* meaning useful) the well-known being the alkaloid Δ^9^ tetrahydrocannabinol (THC). Recent years have seen an explosion of complexity in the field of cannabinoid pharmacology. It was discovered quite early that there were potentially a number of active constituents of the plant and there were two clearly distinguishable receptor subtypes CB_1_ and CB_2_[1, 2], but more recently this list is looking likely to grow, as former so called orphan G-protein coupled receptors such as GPR55[3] and potentially GPR18[4] emerge as receptors for cannabinoids. Furthermore there are well-known ion channels, such as TRPV1 and other proteins such as nuclear peroxisome proliferator-activated receptors (PPAR’s) that appear to be modulated by cannabinoids[5]. This already multifaceted story has started to now become even more complex with the identification of not just agonists, but antagonists, allosteric modulators[6] and inverse agonists[7]. The most basic summary of cannabinoid pharmacology indicates that CB_1_ is generally located to neurones and the CNS[8] and CB_2_ located elsewhere[1]. Naturally every rule has its exceptions. For example many assume that CB_2_ receptors are not expressed in brain, this is in fact inaccurate; although expression of this receptor can be induced in immune cells, they are resident in brain microglia or simply infiltrating immune cells[9]. Cannabinoid receptors are therefore present in the inflammatory pain pathway at both the peripheral and central (spinal and supraspinal) levels[10]. The expression of CB_1_ is largely restricted to neuronal cells and in particular those neuronal cells responsible for nociceptive processing within the brain and the peripheral nervous system[8]. CB_2_ receptor expression is predominantly restricted to immune cells including glia and in the context of this editorial it was originally identified in macrophages[1, 11] (See Figure 1). It is probably too early to be too categorical about the other emerging subtypes.

Enthusiasm for cannabinoids as medicines seems to go through phases. Firstly, it was thought of as a recreational drug; then potential medicinal benefits emerged and later as the widespread (approaching pleiotropic) actions were identified it started to appear as if their actions were too widespread to biomedical and pharmaceutical utility. The discovery of a positive interaction between cannabinoid ligands and TRPV1 was particularly disappointing, since TRPV1 is a widespread mediator of joint nociception[14]. Selective ligands are now emerging however and hope is returning to the field of medicinal cannabinoid research.

## Main text

The separation of CB_1_ and CB_2_ implies that activation of CB_2_ will be without psychotropic effects and so considerable efforts have gone into selective ligands for this receptor in particular. A series of analogues were produced by Huffman *et al*[16] and the paper published by Fukuda *et al* 2014 in BMC Musculoskeletal Disorders[17] explores one of these JWH133 in the context of rheumatology.

Joint pharmacology is an often-overlooked area of research, despite the clear need for novel treatments for a range of disorders. The overall burden of musculoskeletal disease to society is enormous with the majority of elderly people affected. A large part of this is arthritis; the two most common subtypes of which are rheumatoid arthritis (RA), an autoimmune disease that typically progresses to all joints and osteoarthritis (OA), a condition with multiple and less well-defined aetiologies. Although the global prevalence of RA itself is modest (0.24%), the disease is severe and protracted and is therefore a major contributor to pain and disability accounting for approximately 5 million disability-adjusted life years (DALYs) in 2010[18]. In 2002 WHO ranked OA and RA as the first and second largest individual causes of "years lived with disability" (YLD)[19] and the more recent and comprehensive 2010 Global Burden of Disease study placed musculoskeletal disorders as the largest contributor (23.2%) to YLD in the world apart from mental health conditions[20].

Synovitis is increasingly viewed as a pathogenic factor in arthritic diseases. In OA, synovitis is common[13, 21, 22], but in RA it is the central component[23, 24]. A number of potential treatments are available to reduce pain generally (NSAIDs or paracetamol), or inflammation in RA and OA however control of rheumatic pain specifically is difficult. Latest treatments for RA include biological interventions that interfere with TNF-α signalling and the recent discovery of interactions between cartilage and subchondral bone[25] mediated by NGF and the FGF family of peptides has brought some excitement to treatment of OA in particular[26]. Both sprifermin (human recombinant FGF-18) and tanezumab (anti-NFG monoclonal antibody)[27] are both showing promise. Non-peptide drugs are also frequently advantageous over peptides due to their (often) greater ease of preparation and usage. The synovium contains sensory nerve endings however and a clear source of pain; probably in both RA and OA. Whilst a considerable amount is known about the pharmacology of chondrocytes[28], considerably less is known about equally important synovial cells. In fact the synovium as a whole has received less attention that the other joint tissues, partly due to its relative inaccessibility and fragility in a typical rodent model. Interestingly, however, the major cellular component of the normal synovium is type B or fibroblast-like synoviocyte (FLS) and these can be isolated from humans[29] and larger animals[30], but even relatively straightforwardly, from rodents[31]. A well-established contributor of joint inflammation is the infiltration of synovial macrophages. Macrophages express CB_2_ receptors, and additionally, cannabinoid receptors are expressed on neuronal cells. Therefore there is scope for a complex pattern of cannabinoid interactions within the synovium and surrounding joint tissue. Fukoda *et al*[17] now test the efficacy of the 200 fold selective CB_2_ agonist JWH133 against both FLS inflammation and the murine collagen type II (CII)-induced arthritis (CIA) model of RA. They find widespread and encouraging results. *In vitro*, they culture FLS from RA patients and show that FLS produce IL-6, MMP-3, and CCL2 (also known as monocyte chemotactic protein, MCP-1) in response to TNF-α stimulation. This is interesting at a number of levels; IL-6 is known to induce pain[32, 33]. MMP-3 has roles in matrix turn over and may diffuse from the synovium into cartilage in parallel to MMP-13[34]. CCL2 is known to be elevated in RA samples[35], this is a chemokine often referred to as MCP1 and is involved in the recruitment of monocytes, macrophages, T-cells and dendritic cells to the sites of inflammation. Fukoda *et al*[17] also showed how the promising JWH133 was able to inhibit CCL2 expression. There were other observations too. For example, JWH133 inhibited markers of osteoclastogenesis and this too could have implications for preservation of bone loss in RA. Osteoclastogenesis is a multi-complex procedure that includes many stages, and each one of which is a potential therapeutic target in OA[36] and many other diseases including osteoporosis[37]. This is an additional promising role for JWH133 and fits nicely with the observation CB_2_-deficient mice develop osteoporosis with age[37] and that JWH133 attenuated pain behaviours in a rat model of OA[38]. Fukuda *et al* observed[17] an over-all reduction in arthritic "score" in CIA mice injected with JWH133 compared to controls, again enforcing the potential for JWH133.

## Conclusions

Musculoskeletal biology has been in need of a selective CB_2_ agonist in arthritic models. The apparent beneficial effects reported here are comparatively mild, but could be synergistic to each other with benefits in terms of reduced pain, reduced inflammatory activity and reduced osteoclastogenesis of infiltrating macrophages. This is the first report of positive effects of a selective CB_2_ agonist in the CIA model. Although the effect was mild, optimization of dosage and/or treatment protocol might enhance the effect. Perhaps, even more selective CB_2_ agonists might solve this problem. JWH133 is approximately 200 fold selective (CB_2_/CB_1_)[16] and the future may see selectivity orders of magnitude greater than this in the near future. So this study is early; but very encouraging, providing yet more evidence[39] that the therapeutic potential of cannabis extracts, and its analogues are enormous.

## Authors’ information

AM and RBJ are members of the D-BOARD European Consortium for Biomarker Discovery

http://www.d-board.eu/dboard/index.aspx

http://cordis.europa.eu/projects/rcn/105314_en.html

http://ec.europa.eu/research/health/medical-research/severe-chronic-diseases/projects/d-board_en.html

## Authors’ original submitted files for images

Below are the links to the authors’ original submitted files for images. Authors’ original file for figure 1

## Abbreviations

CIA: Collagen type II (CII)-induced arthritis
DALY: Disability-adjusted life years
FLS: Fibroblast-like synoviocytes
MMP: Matrix metalloproteinase
MCP-1: Monocyte chemotactic protein
NSAIDs: Non-steroidal anti-inflammatory drugs
OA: Osteoarthritis
PPAR: Peroxisome proliferator-activated receptor
RA: Rheumatoid arthritis
THC: Δ^9^ tetrahydrocannabinol
YLD: Years lived with disability.
