# Supplementary figures and images for: High hopes for cannabinoid agonists in the treatment of rheumatic diseases
Source: BMC Musculoskelet Disord. 2014 Dec 4;15:410. doi: 10.1186/1471-2474-15-410 (PMC4289380; doi:10.1186/1471-2474-15-410)

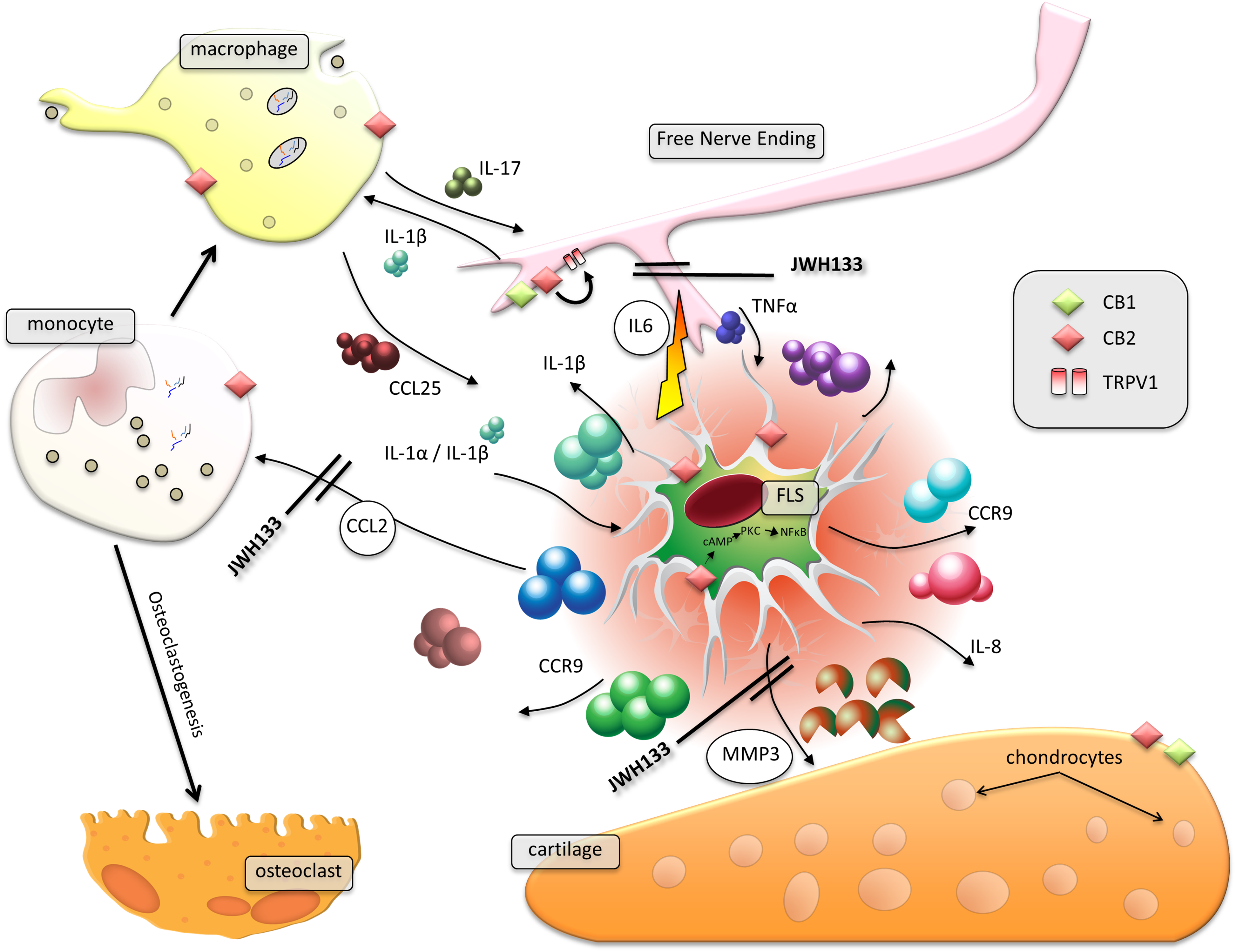

Supplement: Supplementary file 1 — Authors’ original file for figure 1 [file 12891_2014_2353_MOESM1_ESM.tif]
